# Evaluation of feasibility and acceptability of a web-based diabetes prevention program (DPP) for diabetes risk reduction in Chinese Americans in New York City

**DOI:** 10.3389/fpubh.2023.1199746

**Published:** 2023-06-02

**Authors:** Ming-Chin Yeh, Wincy Lau, Claire Anselmo Keady, Margrethe Horlyck-Romanovsky, Ho-Jui Tung, Lu Hu, Grace X. Ma, Judith Wylie-Rosett

**Affiliations:** ^1^Nutrition Program, School of Urban Public Health, Hunter College, City University of New York, New York, NY, United States; ^2^Department of Health and Nutrition Sciences, Brooklyn College, City University of Brooklyn, New York, NY, United States; ^3^Department of Health Policy and Community Health, Jiann-Ping Hsu College of Public Health, Georgia Southern University, Statesboro, GA, United States; ^4^Department of Population Health, NYU Grossman School of Medicine, New York, NY, United States; ^5^Center for Asian Health, Lewis Katz School of Medicine, Temple University, Philadelphia, PA, United States; ^6^Department of Epidemiology and Population Health, Albert Einstein College of Medicine, Bronx, NY, United States

**Keywords:** Chinese Americans, web-based diabetes prevention program, cultural and linguistic adaptation, feasibility, acceptability, qualitative study

## Abstract

**Introduction:**

Intensive lifestyle intervention remains an effective modality to reduce diabetes incidence and delay the progression to type 2 diabetes. The primary aim of this study was to pilot-test the feasibility and acceptability of a culturally and linguistically tailored web-based DPP intervention among Chinese Americans with prediabetes living in New York City.

**Methods:**

Thirteen Chinese American participants with prediabetes were recruited to complete a 1-year web-based Diabetes Prevention Program (DPP) lifestyle intervention. Quantitative and qualitative measures such as retention rate and data collected from web-based questionnaires and focus groups were collected and analyzed to assess study feasibility and acceptability.

**Results and Discussion:**

Participants were receptive to the program through high engagement, retention and satisfaction. Retention rate was 85%. 92% of participants completed at least 16 sessions out of 22 sessions. Post-trial surveys indicated high satisfaction of 27.2/32 based on Client Satisfaction Questionnaire (CSQ-8) score. Participants expressed the program increased their knowledge and methods to prevent onset of type 2 diabetes such as incorporating healthy eating habits and increasing physical activities. Although not a primary outcome, there was a significant weight reduction of 2.3% at the end of month 8 of the program (*p* < 0.05). The culturally and linguistically adapted DPP *via* online platform successfully demonstrated feasibility and acceptability among Chinese Americans with prediabetes. Further evaluation of the web-based Chinese Diabetes Prevention Program in a larger trial is warranted.

## 1. Introduction

In 2022, 96 million US adults (38% of US population) were estimated to have prediabetes, and 37.3 million people had diabetes (1). Intensive lifestyle intervention remains an effective modality to reduce diabetes incidence and delay the progression to type 2 diabetes. The Diabetes Prevention Programs (DPPs) which initiated in 1996 have shown lifestyle intervention effectively reduce incidence of type 2 diabetes by 58% when compared to placebo in randomized controlled trials (2). Since then, in-person DPPs were successfully implemented in various community and clinical settings (3–5). However, barriers associated with in-person DPPs include limited access of transportation, scheduling difficulties and discomfort feelings in group settings (6). The COVID-19 pandemic has limited in-person contacts and exacerbated the situation. Data show that the COVID-19 pandemic has stimulated telehealth services dramatically, from 2.1 million to 32.5 million with March 2020 to February 2021, according to the U.S. Government Accountability Office (7). Telehealth use under Medicare also increased tenfold in 2020 (7). Technology-assisted DPP programs may provide an alternative option and increased reach for prediabetes and underserved populations who experience barriers to participate in disease prevention programs. (8–10). Online DPPs have demonstrated a higher participation, significant weight loss and improvement in HbA1c when compared to in-person DPP (8, 11).

Leveraging the success of our team’s prior research on developing a culturally and linguistically tailored DPP curriculum using Community-Based Participatory Research and Intervention Mapping approaches (12), we have developed an online DPP program in the hopes of increasing access of diabetes prevention programs among Chinese Americans with prediabetes (13, 14). Therefore, the primary aim of this study was to assess the feasibility and acceptability of a culturally and linguistically tailored web-based DPP intervention among Chinese Americans with prediabetes living in New York City.

## 2. Materials and methods

### 2.1. Study design

This was a pilot study based on an adapted DPP program the research team has developed; a detailed development process of the online curriculum and protocol was published earlier (14). Briefly, the program was a single arm design, and duration is 1 year, which begins with a 16-week core phase and followed by six monthly maintenance phase. In addition, two focus groups were conducted (one at the end of the 16-week intervention and the other at the end of the 6-month post intervention) to explore the feasibility and acceptability of the adapted program. Mixed-methods approaches were used to collect data at baseline and throughout the intervention utilizing both quantitative and qualitative methods.

### 2.2. Study participants

Chinese Americans with prediabetes were recruited from New York City. A purposive sample of study participants were recruited through physician referrals and community health fairs. The inclusion criteria for study participants included Chinese Americans with prediabetes [HbA1c 39–46 mmol/mol (5.7–6.4%)], a body mass index (BMI) ≥ (22) kg/m^2^, Chinese speaking and a willingness/ability to provide informed consent; and accessibility to web-based tools.

### 2.3. Online DPP intervention

Eligible participants were invited to attend an online orientation prior to the start of the intervention. During the orientation, participants were informed about the study procedures, and a signed informed consent was obtained from all study participants. They were assisted in setting up a Facebook account under a pseudonym. Participants’ weight and height for BMI and demographic survey were collected at the beginning of the study. They were provided a digital pedometer as non-monetary compensation to record their steps throughout the study. They were also provided with a user manual on how to navigate weekly modules and online homework on Facebook and Qualtrics.

The online DPP modules were housed in a private Facebook group that is accessible only to study participants and research staff; program modules were delivered as photos in the weekly/monthly posts. A typical weekly module included reading materials and two Qualtrics links for self-monitoring data collection; one for online homework and the other for food intake and step records logbook (14). A new module was posted to Facebook weekly. The curriculum is summarized in Table 1. Questions in the online homework included open-ended questions related to the corresponding modules, as well as rating each module’s clarity and cultural/linguistic appropriateness of the content for Chinese Americans. Each module contained 6 to 10 pages of reading materials that takes approximately 20 min to review. It takes another 15–20 min to complete the homework. The web-based DPP program was conducted between April 2021 and March 2022.

**Table 1:** Summary of DPP Curriculum for Chinese Americans.

Session	Modules in English	Modules in Chinese
*Weekly core modules*		
1	Introduction to the Program	課程介紹
2	Get active to prevent T2	保持活躍，預防二型
3	Track your activity	記錄你的活動
4	Eat well to prevent T2	食得健康，預防二型
5	Track your food	記錄你的飲食
6	Get more active	增加活動
7	Burn more calories than you take	少吃多動，消耗熱量
8	Shop and cook to prevent T2	購物烹調，預防二型
9	Manage Stress	應付壓力
10	Find time for fitness	善用時間，活動健身
11	Cope with triggers	應對慣性行為
12	Keep your heart healthy	保持心臟健康
13	Take charge of your thoughts	保持正面想法
14	Get support	爭取支持
15	Eat well away from home	外出用餐，健康進食
16	Stay motivated to prevent T2	積極堅持，預防二型
*Monthly post-core modules*		
17	When weight loss stalls	減重大計，停滯不前
18	Take a fitness break	多作健身運動
19	Stay active to prevent T2	持續活躍，預防二型
20	More about carbs	了解碳水化合物了解
21	Have healthy food you enjoy	盡情享受，健康食物
22	Prevent T2 for life	預防二型，終身受用

As mentioned above, we also conducted two online focus groups after the 16-week core phase to refine the online program and after the 1-year intervention for program sustainability. Both focus groups were held in Cantonese and Mandarin by WL *via* Zoom (Zoom Video Communications, Inc., San Jose, CA). The focus groups were audio-taped and lasted approximately 1 hour. The focus groups were conducted in October 2021 and April 2022. Questions for the two focus groups focused specifically on the feasibility and acceptability of the online program. Focus group guides can be found in Supplementary material. At the end of the study, participants could receive up to USD $120 monetary compensation if they completed the 1-year program and two focus groups. The study was approved by the Institutional Review Board of Hunter College, City University of New York (protocol #2018–1,008).

### 2.4. Measurements

*Participant characteristics.* Demographic information was collected at baseline: age, education, employment, annual household income, relationship status, internet access and accessibility of internet tools.

#### 2.4.1. Intervention feasibility

##### 2.4.1.1. Engagement

Retention rate was used to assess level of engagement. Participants received text reminders by a lifestyle coach weekly to complete the modules and homework. Module views and completeness of online homework were collected and analyzed to assess feasibility. Retention rate was calculated based on participants who had viewed all of the 1-year modules and completed the online homework.

##### 2.4.1.2. Usability

Upon completion of each module, participants were asked to rate the modules’ clarity on a 5-point Likert scale. They were also asked whether they considered the content culturally and linguistically appropriate on a 7-point Likert scale. Participants were further asked to provide feedback and comments and state any technical issues with each module. Finally, participants were asked to rate how likely they are willing to participate in future studies in a web-based format at the end of the intervention. To facilitate social support, an additional Facebook private discussion group was created for participants to share healthy lifestyle information and act as a support group.

#### 2.4.2. Intervention acceptability

##### 2.4.2.1. Usefulness

At the end of the core phase, participants were asked whether they liked this online diabetes prevention program (DPP) and whether they thought this DPP program helped them engage in a healthy lifestyle such as eating healthy and/or being active with optional comments. In addition, they were asked how likely they were willing to participate future studies in web-based format. They were also encouraged to utilize self-monitoring tools (uploading meal photos and step records using the provided digital pedometer).

##### 2.4.2.2. Satisfaction

The Client Satisfaction Questionnaire (CSQ-8) (15) was used to assess intervention satisfaction. The CSQ-8 was adapted for this study by substituting “service” with “help” and “program” with “DPP.” The overall sum ranged from 8 to 32 with a higher score indicating higher satisfaction.

#### 2.4.3. Secondary outcomes

To evaluate the efficacy of the online DPP curriculum, the weights of participants were obtained at baseline and self-reported weight measurements were collected at month 4, month 8, month 10 and month 12 of the intervention.

### 2.5. Analysis

#### 2.5.1. Qualitative analysis

Qualitative data were collected from web-based questionnaires throughout the intervention and at the end the core phase (month 4) and maintenance phase (month 12) which contains a post intervention survey and were used to evaluate feasibility and acceptability of the intervention. Qualitative data were also collected from two focus group discussions after the 16-week core phase and after the 1-year intervention. The qualitative data were analyzed by reviewing and consolidating transcripts by the research team to summarize major themes. Specifically, analysis involved initial open coding of text, which was done by a team member (WL) independently using ATLAS.ti (RRID:SCR_022920) (16) then modified by reading transcripts repeatedly to resolve coding differences. A second team member (MCY) would review codes and themes to provide consensus for study findings. An inductive approach was used to define the themes (17). Reflexivity considerations such as cultural background, linguistic tradition, personal preferences, and social position were observed, especially during data collection and analysis, to reduce potential bias (18, 19).

#### 2.5.2. Quantitative analysis

For quantitative data (e.g., multiple-choice questions), statistical analyses were performed with IBM SPSS Statistics (RRID:SCR_019096). We used descriptive statistics to summarize demographics among all study participants. Descriptive statistics were used to describe DPP feasibility and acceptability and study feasibility. Paired samples *t* test was performed to examine weight changes between baseline and post-intervention. Given the small sample size of this study, 95% confidence intervals were used for differences in means.

## 3. Results

### 3.1. Participant characteristics

Table 2 presents the demographic characteristics of all study participants. Participants’ ages ranged from 57 to 74 years, and the mean age was 66 ± 5.8 years. The majority (85%) of the participants were female, more than half (54%) of participants were married, and more than half (61%) of participants reported being retired. Fewer than half of participants (23%) had post-secondary and higher education. All participants had their own smartphones with internet access. The majority of participants (85%) had access to two or more technological devices including computers, laptops, tablets and smartphones.

**Table 2:** Demographic and characteristics of study participants (*n* = 13).

Characteristic	
Age, mean (SD)	66 (5.8)
Sex, female, (*n,* %)	11 (85)
Education, (*n,* %)	
Below high school graduate	4 (31)
High school graduate	4 (31)
College graduate	3 (23)
Prefer not to say	2 (15)
Employment status, (*n,* %)	
Employed Full-Time	1 (8)
Employed Part-Time	1 (8)
Retired	8 (61)
Prefer not to say	3 (23)
Household income, (*n,* %)	
Less than $25,000	3 (23)
$25,000–$50,000	2 (15)
$50,000–$100,000	1 (8)
Prefer not to say	7 (54)
Marital status, (*n,* %)	
Married	7 (54)
Single	3 (23)
Widowed	1 (8)
Prefer not to say	2 (15)
Internet Access	13 (100)
No. of technological devices (e.g., phone, iPad)	
More than 2	11 (85)

### 3.2. Intervention feasibility

During the pilot feasibility study, 19 potentially eligible participants were approached. Fifteen participants were screened to be eligible to participate the study, 13 of whom (86.7%) were enrolled into the study. Two participants were excluded due to withdrawal of the program at week 2 and after week 16 due to busy schedules and waning interest the study.

#### 3.2.1. Engagement

By the end of week 16 (core phase), 12 of the 13 participants viewed and completed all 16-week DPP materials. This resulted in a retention rate of 92% at week 16. And by the end of the 1-year program, 11 of the 13 participants viewed and completed all 1-year DPP materials. This resulted in a retention rate of 85% at 1-year interval. It is observed that the core phase of the intervention (Module 1–16) had a higher completion rate. The completion rate slightly declined in the maintenance phase of the intervention (Modules 17–22).

#### 3.2.2. Usability

Overall, participants reported that the weekly modules were clear and understandable. Based on a 5-point Likert scale, module 3 on Track Your Activity, module 8 on Shop and Cook to Prevent T2 and module 15 on Eat Well Away from Home were reported as most clear and understandable (mean score of 4.6 ± 0.5), while module 2 on Get Active to Prevent T2 as least clear and understandable (mean of 4.0 ± 1.3). Most of the modules were considered culturally and linguistically appropriate (6.0 ± 0.9) based on 7-point Likert scale. Out of 11 respondents who completed the 1-year program, seven (64%) reported that they are likely/very likely to participate in future studies in web-based format. For social support, only two participants (15%) used the private discussion group created in Facebook and shared exercise video.

### 3.3. Intervention acceptability

#### 3.3.1. Usefulness and satisfaction

The program was well-received by the participants. All (100%) of the respondents reported that they liked the online DPP and agreed this program helped them engage in a healthy lifestyle. The self-monitoring tools were highly accepted by the participants as 12 of 13 (92%) participants utilized the features to upload three days’ of meal photos and step records 12 or more weeks. Participants were also quite satisfied with the program as the average intervention satisfaction CSQ-8 score was 27.2 ± 2.5 on a range of 8–32.

### 3.4. Secondary outcomes

Using paired *t* tests, there were significant differences in mean weight changes when comparing with month 2, month 3, month 4, month 8 and month 10 to baseline (see Table 3). However, the significance was not sustained at the end of 12 months.

**Table 3:** Mean weight change over 1 year.

	Weight, kg (mean ± SD)	Weight change from baseline Mean (95% CI)	%
Baseline	63.4 ± 11.4	–	–
Month 1	63.2 ± 11.6	0.28 (−0.15, 0.70)	−0.44
Month 2*	62.8 ± 11.2	0.64 (0.01, 1.27)	−1.00
Month 3*	62.4 ± 11.3	1.00 (0.30, 1.70)	−1.58
Month 4*	62.3 ± 11.5	1.13 (0.34, 1.91)	−1.78
Month 8*	63.4 ± 11.4	1.45 (0.23, 2.68)	−2.29
Month 10*	63.5 ± 11.1	1.30 (0.04, 2.56)	−2.00
Month 12	63.8 ± 11.9	1.03 (−0.46, 2.52)	−1.62

*p < 0.05, compared to baseline.

### 3.5. Qualitative results

Eleven participants joined focus group 1 at the end of the core phase intervention, and 10 participants participated in focus group 2 at the end of 1 year program. The thematic summary of the focus group findings on the feasibility and acceptability of the online DPP program are outlined below.

#### 3.5.1. Intervention feasibility

In terms of usability, several participants reported that they encountered difficulties in the first 2 weeks of the program due to low online literacy (for example, one participant stated *“It was my first time, so it was quite difficult to operate,”* while some participants stated that the curriculum was easy to understand with comments such as *“The contents of the curriculum are easy to understand, vivid and abundant”*).

Participants also shared different opinions on food intake record. Some participants expressed that they learned the My Plate method from DPP to keep track of a balanced meal and portion sizes. Many expressed that it was difficult for them to record their intake at each meal because in Chinese culture meals are often shared in family style without individual portions, making portion sizes difficult to track. Here are two common sentiments: *“Chinese food is more difficult [to track], Western food is easier [to track]” and “I rarely use plates to divide one by one.”*

Although the majority of the participants expressed that the digital pedometer was a great tool to keep track of physical activity, surprisingly, many preferred using their own phone over a pedometer. One participant stated that *“Smartphone is more convenient because it is always around.”*

The social support group on Facebook demonstrated low participation with only 2 participants posting videos and commented in the private discussion group. Participants expressed that they were not familiar with the features of Facebook and would be familiar with texting software popular among Chinese immigrants such as WeChat.

#### 3.5.2. Intervention acceptability

Consistent with quantitative findings shown earlier, focus group findings also illustrated that participants generally were highly receptive to the web-based 1-year program. Most participants shared the same experiences of learning more in-depth information about preventing progression of type 2 diabetes and they liked recording diet and physical activity using photos and digital pedometer.

The majority of the participants preferred online learning over in-person classes as online classes allow flexibility with their schedules. For example, *“I prefer online learning because you can manage your own schedule, like in the past, if the class location is not close to home, you will have to commute around. The time is flexible and homework can be done in the morning or evening. Like today’s Zoom, if you want to walk to Chinatown, you will waste a lot of time. I agree with this approach now” and “The program is convenient, and we lived far away, 4 h round trip.”*

Participants also revealed that the COVID-19 pandemic helped them achieve healthier eating habits. For example, they reduced their frequency of eating out and learned how to eat healthier. Many also learned making a shopping list before going to the grocery store: “*Nowadays I always have a shopping list before I go to the grocery store.”*

#### 3.5.3. Improvements

Participants also provided valuable insights to improve the program, including technical issues such as allowing participants to revisit their homework and saving unfinished surveys to prevent repetitive work. In regard to programmatic issues, the majority of participants preferred bi-weekly sessions over monthly sessions in the maintenance phase. As one participant put it: *“Two weeks seems better, because you know that the older adult will forget after too long.”* Moreover, most participants suggested one-on-one follow up sessions during the monthly maintenance phase as they prefer individualized reinforcements and feedbacks from lifestyle coach.

## 4. Discussion

This pilot study demonstrated feasibility and acceptability of the translated DPP curriculum delivered through a web-based format. To our knowledge, this is the first feasibility study assessing a web-based DPP using a culturally and linguistically appropriate curriculum among Chinese Americans with prediabetes. The program was well perceived with 85% of study participants completing all 22 modules (16 core weekly modules plus six-monthly modules) and homework over 1-year. The high level of engagement is consistent with other feasibility studies that delivered digital diabetes prevention programs. For example, a study conducted a telehealth-adapted DPP at a senior center for 6 weeks demonstrated 80% attendance rate and 75% retention rate, which had a similar retention rate with our study (20). Fontil et al. (21) assessed the feasibility of an existing digital health DPP adapted for low-income prediabetes patients. The result established a high engagement rate with 80% of participants logged in at least once/week. Our study generated an overall 85% of participants who logged in at least once a week and completed the homework, suggesting high feasibility of the 1-year program. The high level of engagement may be explained by weekly contact between lifestyle coach and participants which served as a motivation support and facilitated behavior change (22).

The web-based DPP was highly acceptable and was rated as useful. Participants reported overall satisfaction based on usability and content. They expressed the language in the curriculum was culturally and linguistically appropriate. This is in line with our previous studies using a translated DPP curriculum for an in-person DPP (12) Surprisingly, despite barriers related to online literacy reported in the first 2 weeks of intervention, participants demonstrated high rates of completion and remained engaged until the end of 1-year program. This suggested that technical difficulties did not affect usability of the digital intervention, which is also in line with a digital DPP conducted in low-income patients in Southern California (9). It is suggested digital literacy was not a major barrier for people who want to improve their health (9). In the current study, we speculated that this barrier was overcome by providing a manual with step-by step instructions during orientation and *via* subsequent interactions with our lifestyle coach throughout the intervention.

Our study, although not powered to detect significant changes in weight and lacking a comparison group, showed that there was significant weight reduction at month 2 (−1%), month 3 (−1.58%), month 4 (−1.78%), month 8 (−2.29%) and month 10 (−2%) when compared to baseline. This small, yet significant result demonstrated that the culturally and linguistically tailored web-based DPP shows promising effects on weight loss, especially during the core phase. Moreover, although the standard DPP recommends a 7% weight loss goal, evidence showed that there were variations by race in achieving this goal. (23) It is reported that while 68% of white participants achieved ≥5% weight loss at 12 months, only 64% of Asian participants did (23) suggesting the weight loss goal should be adjusted for different race, especially as Asians having a potentially lower cut off point for overweight (24).

Standard DPP program offers monthly maintenance sessions (25). Surprisingly, according to our focus group findings, participants prefer bi-weekly to monthly maintenance sessions. This is particularly interesting as minority populations tend to have less motivation to participate and engage in health promotion programs due to a busy work schedule, distrust of medical establishment, health literacy and family composition (26–28). It is possible that the request for more frequent meeting times we observed is because the study was conducted at the height of the COVID-19 pandemic (29) and people were craving for more contacts (29). It remains to be seen if this trend continues post pandemic as it could have implications for the scheduling of future DPP programs.

Despite the high engagement in our DPP program, the low utilization of the social support group on Facebook suggested that online social support interaction seemed to contribute little to program completion and satisfaction. Our participants stated the major reason of not utilizing the discussion group was because they were not familiar with the features of Facebook. We also suspect that participants viewed the social support group as an optional activity. For example, a family-based adolescent substance abuse prevention program with high engagement using Facebook as an additional support also encountered challenges in involving participants to interact online (30). It is suggested that providing opportunities for social support may not help improving program engagement due to being highly reliance on group member’s skills, trust and sense of attachment to the group (30).

Several features distinguish our web-based DPP from other traditional DPPs. First, our pilot web-based DPP successfully delivered the contents of a 1-year DPP intervention asynchronously without the need of participants to attend in-person classes often seen in traditional DPPs. The flexibility of time may have contributed to a higher participation rate which has also been shown in other web-based DPPs when compared to in-person DPPs (11). Our pilot study also demonstrated the efficacy of self-learning from online modules in facilitating healthy lifestyle changes such as learning about strategies of healthy eating and increasing physical activities to improve health outcomes without the need for an on-site instructor in traditional DPPs. This approach could not only save valuable program resources but also enhance the program’s reach to often hard-to-reach participants such as men or racial/ethnic minorities to engage in health promotion programs.

There are several strengths of this feasibility study, including offering an online diabetes prevention program for a rapidly growing minority population, testing the feasibility of the program for a full 1-year that includes core and post-core modules, and collecting both quantitative and qualitative outcomes. Nonetheless, our study has several limitations. First, this is a pilot study with a very small sample size and findings are only generalizable to people who are highly engaged. Second, features such as built-in messages or feedback loops were limited in the current online version. The lack of a fully automated function may impact its feasibility as an asynchronous online curriculum. Third, we were able to collect only self-reported weights post-intervention due to the COVID-19 pandemic, which may contribute to errors and biases in weight outcomes. Furthermore, due to funding constraints, we were not able to collect blood samples to obtain objective measures such as A1c from study participants.

## 5. Conclusion

In summary, this study demonstrated feasibility and acceptability through engagement, satisfaction and significant weight reduction for delivering an online diabetes prevention program among Chinese Americans with prediabetes. Recommendations for improvements for future studies include enhancing communications between lifestyle coaches and participants by sending automated messages regularly, finding other means to promote social support discussions; and adding automated feedback loops to enhance the functionality of online programs. In addition, replication of these findings in a larger study is warranted.

## Data availability statement

The original contributions presented in the study are included in the article/Supplementary material, further inquiries can be directed to the corresponding author.

## Ethics statement

The studies involving human participants were reviewed and approved by Institutional Review Board of Hunter College, City University of New York. The patients/participants provided their written informed consent to participate in this study.

## Author contributions

M-CY conceptualized the study, drafted the focus group discussion guides, interpreted the findings, drafted the manuscript outline, and contributed to the writing of the manuscript. WL assisted in facilitating focus group discussion, transcribing focus group texts, and reviewing transcripts, helped interpret the findings, and contributed to the writing of the manuscript. CK, MH-R, H-JT, LH, GM, and JW-R provided feedback to the focus group discussion guides and reviewed and provided feedback to drafts of the manuscript. All authors contributed to the article and approved the submitted version.

## Funding

This research was funded by PSC-CUNY Award # 61707–0049 and NIH/NIGMS award # 5SC3GM131949–02 (PI: M-CY) and, in part, by City University of New York Interdisciplinary Research Grant 2022–2023 #8020905–26 (PI: MH-R), TUFCCC/HC Regional Comprehensive Cancer Health Disparity Partnership, Award # U54 CA221704(5) from NIH/NCI (Contact PIs: GM and Ogunwobi) and by the NY-Regional Center for Diabetes Research l (grant # DK111022), which supported the effort of JW-R. The contents of this manuscript are the responsibility of the authors and do not necessarily represent the official views of the NIGMS/NIH. NCI/NIH or NIDDK/NIH.

## Conflict of interest

The authors declare that the research was conducted in the absence of any commercial or financial relationships that could be construed as a potential conflict of interest.

## Publisher’s note

All claims expressed in this article are solely those of the authors and do not necessarily represent those of their affiliated organizations, or those of the publisher, the editors and the reviewers. Any product that may be evaluated in this article, or claim that may be made by its manufacturer, is not guaranteed or endorsed by the publisher.
