# Supplementary material for: Evaluation of feasibility and acceptability of a web-based diabetes prevention program (DPP) for diabetes risk reduction in Chinese Americans in New York City
Source: Front Public Health. 2023 Jun 2;11:1199746. doi: 10.3389/fpubh.2023.1199746 (PMC10272575; doi:10.3389/fpubh.2023.1199746)
Supplement: Supplementary file 1 [file Data_Sheet_1.docx]

Supplementary Material

# Supplementary Data

Online DPP intervention focus group questions at 6 months

**Intervention feasibility**

1. What did you think of the information presented in the online DPP curriculum?

Probe: [Amount of information, length of presentation]

2. How much of this information was new to you? If it was not new to you, where have you heard the information in the past?

3. Which method did you use most to track your food intake?

Probe: [phone applications/ hand-written food diary/ send photos to RA/taking photos and uploading it on Qualtrics.]

4. Which method did you use most to track your physical activity?

Probe: [phone applications/ digital bracelet/ hand-written journal]

5. What did you do when you receive educational material that was confusing or unclear?

Probe: [Did you talk to RA through phone counseling or text messages/ Did you search information online yourself or asked your family/ group members?]

6. If we have to make one change that would make the 6-month online DPP curriculum better in the future, what could we do to improve it?

**Intervention acceptability**

1. What did you like the best about the program so far?

Probe: [General/ amount of information, activity, time commitment, convenience]

2. What did you like the least about the program so far?

Probe: [General/ amount of information, activity, time commitment, convenience]

3. What were the goals you have set when enrolling in this program? Did you achieve your goals? If yes, what did you do to achieve the goals? If no, what went wrong?

Probe: [Increasing physical activity/ improving diet/ weight monitoring/ engaging family or social support/ other barriers]

4. Did you feel the goals you have set were too high or too low?

5. What was the hardest/easiest thing you had to do to achieve these goals?

[Increasing physical activity/ improving diet/ engaging family or social support]

6. If you joined the private peer support group on Facebook, what did you like or did not like about the interaction with other participants?

7. Will you continue to participate in the 6-month post intervention? Why or why not?

**Intervention feasibility**

1. What did you think of the information in the online DPP curriculum?

Probe: [Amount of information, length of presentation]

2. How much of this information was new to you? If it was not new to you, where have you heard the information in the past?

3. Which method did you use most to track your food intake?

Probe: [phone applications/ hand-written food diary/ send photos to lifestyle coaches/taking photos and uploading it on Qualtrics.]

4. Which method did you use most to track your physical activity?

Probe: [phone applications/ digital bracelet/ hand-written journal]

5. If you stopped tracking your food intake and/or physical activity, what made you stop? What could the research team have done differently to help you keep track of your progress?

6. What did you do when you receive educational material that was confusing or unclear?

Probe: [phone counseling/ text messages/ search on internet/ ask family/ friends]

7. Do you like the 6 monthly post-intervention sessions? Why or why not?

8. If we have to make one change that would make the 1-year online DPP curriculum better in the future, what could we do to improve it?

Online DPP intervention focus group questions at 12 months

**Intervention acceptability**

1. What did you like the best about the program?

Probe: [General/ amount of information, activity, time commitment, convenience]

2. What did you like the least about the program?

Probe: [General/ amount of information, activity, time commitment, convenience]

3. We know that you are interested in learning about healthy lifestyle through technology because you participated in our online DPP program. Do you see your family/friends participating in a technology-based program like this? If not, why not?

4. What were the goals you have set when enrolling in this program? Did you achieve your goals? If yes, what did you do to achieve the goals? If no, what went wrong?

Probe: [Increasing physical activity/ improving diet/ weight monitoring/ engaging family or social support/ other barriers]

5. Did you feel the goals you have set were too high or too low?

6. What was the hardest/easiest thing you had to do to achieve these goals?

Probe: [Increasing physical activity/ improving diet/ engaging family or social support]

7. If you joined the private peer support group on Facebook, what did you like or did not like about the interaction with other participants?

8. If this program were repeated, what would you like to be changed?

9. Finally, is there anything else you want to share with us?
